# Primary immunodeficiency associated with chromosomal aberration – an ESID survey

**DOI:** 10.1186/s13023-016-0492-1

**Published:** 2016-08-02

**Authors:** Ellen Schatorjé, Michiel van der Flier, Mikko Seppänen, Michael Browning, Megan Morsheimer, Stefanie Henriet, João Farela Neves, Donald Cuong Vinh, Laia Alsina, Anete Grumach, Pere Soler-Palacin, Thomas Boyce, Fatih Celmeli, Ekaterini Goudouris, Grant Hayman, Richard Herriot, Elisabeth Förster-Waldl, Markus Seidel, Annet Simons, Esther de Vries

**Affiliations:** 1 Department Pediatrics, Jeroen Bosch Hospital, P.O. Box 90153, 5200 ME ‘s-Hertogenbosch, The Netherlands; 2 Department of Pediatrics, Amalia Children’s Hospital and Radboud Institute for Molecular Life Sciences, Radboudumc, Nijmegen, The Netherlands; 3 Immunodeficiency Unit, Inflammation Center and Center for Rare Diseases, Children’s Hospital, Helsinki University and Helsinki University Hospital, Helsinki, Finland; 4 University Hospitals of Leicester NHS Trust, Leicester, UK; 5 Children’s Hospital of Philadelphia, Philadelphia, USA; 6 Primary Immunodeficiencies unit Hospital Dona Estefania, Centro Hospitalar de Lisboa Central, Lisbon, Portugal; 7 McGill University Health Centre, Montreal, Canada; 8 Allergy and Clinical Immunology Department, Hospital Sant Joan de Deu, Barcelona, Spain; 9 Faculty of Medicine ABC, São Paulo, Brazil; 10 Pediatric Infectious Diseases and Immunodeficiencies Unit, Hospital Universitari Vall d’Hebron, Barcelona, Spain; 11 Mayo Clinic, Rochester, Minnesota USA; 12 Antalya Education and Research Hospital Department of Pediatric Immunology and Allergy, Antalya, Turkey; 13 Universidade Federal do Rio de Janeiro, Rio de Janeiro, Brazil; 14 Epsom & St Helier University Hospitals NHS Trust, Carshalton, UK; 15 NHS Grampian, Aberdeen, Scotland; 16 Department of Pediatrics and Adolescent Medicine, Center for Congenital Immunodeficiencies, Medical University Vienna, Wien, Austria; 17 Pediatric Hematology-Oncology, Medical University Graz, Graz, Austria; 18 Department of Human Genetics, Radboudumc, Nijmegen, The Netherlands; 19 Department Tranzo, Tilburg University, Tilburg, The Netherlands

**Keywords:** Genetics, Immunology, Chromosomal aberration, Primary immunodeficiency, Mental retardation, Recurrent infections

## Abstract

**Background:**

Patients with syndromic features frequently suffer from recurrent respiratory infections, but little is known about the spectrum of immunological abnormalities associated with their underlying chromosomal aberrations outside the well-known examples of Down and DiGeorge syndromes. Therefore, we performed this retrospective, observational survey study.

**Methods:**

All members of the European Society for Immunodeficiencies (ESID) were invited to participate by reporting their patients with chromosomal aberration (excluding Down and DiGeorge syndromes) in combination with one or more identified immunological abnormalities potentially relating to primary immunodeficiency. An online questionnaire was used to collect the patient data.

**Results:**

Forty-six patients were included from 16 centers (24 males, 22 females; median age 10.4 years [range 1.0–69.2 years]; 36 pediatric, 10 adult patients). A variety of chromosomal aberrations associated with immunological abnormalities potentially relating to primary immune deficiency was reported. The most important clinical presentation prompting the immunological evaluation was ‘recurrent ear-nose-throat (ENT) and airway infections’. Immunoglobulin isotype and/or IgG-subclass deficiencies were the most prevalent immunological abnormalities reported.

**Conclusions:**

Our survey yielded a wide variety of chromosomal aberrations associated with immunological abnormalities potentially relating to primary immunodeficiency. Although respiratory tract infections can often also be ascribed to other causes (e.g. aspiration or structural abnormalities), we show that a significant proportion of patients also have an antibody deficiency requiring specific treatment (e.g. immunoglobulin replacement, antibiotic prophylaxis). Therefore, it is important to perform immunological investigations in patients with chromosomal aberrations and recurrent ENT or airway infections, to identify potential immunodeficiency that can be specifically treated.

**Electronic supplementary material:**

The online version of this article (doi:10.1186/s13023-016-0492-1) contains supplementary material, which is available to authorized users.

## Background

‘Syndromic’ patients frequently suffer from recurrent respiratory infections; it is a major cause of morbidity and mortality in this patient group. However, in these patients immunological work-up is often not performed because an immunodeficiency is not suspected. The infections are often ascribed to food and saliva aspiration [1], structural abnormalities of the upper respiratory tract, neuromuscular problems, malnutrition or institutionalization. Besides, other problems are often more prominent than the recurrent infections. This may lead to underdiagnosis of ‘syndromic immunodeficiency’. However, identification of an underlying immune defect may be therapeutically actionable, which in turn may improve the quality of life in these patients: for instance hypogammaglobulinemia can be treated with immunoglobulin replacement [2, 3]. In addition, information regarding genes critical for the development and functioning of the immune system may be gained by analyzing the precise chromosomal defect and the concomitant immunological phenotype.

Several primary immunodeficiency (PID) disorders have been identified and increasingly their genetic backgrounds have been unraveled [4]. Syndromes with chromosomal abnormalities of number or structure are considered as a distinct group within PID [5]. Clear examples are Down syndrome (trisomy 21) [6] and DiGeorge syndrome (22q11 deletion) [7]. Also, Turner syndrome [8] and Wolf-Hirschhorn syndrome [9] are known to be associated with immunodeficiency. In the past ten years, thirteen cases, three patient series and two families with other chromosomal aberrations and immunological abnormalities have been described in the literature [10–27]. There is one study that screened patients with dysmorphic disorders for immune defects. They showed a high incidence of immunodeficiency in this population (23 out of 29 patients had one or more defects); however, they also included 11 patients with Down syndrome [28]. We hypothesize that in patients with chromosomal aberrations, other than the well-known Down and DiGeorge syndromes, concomitant ‘syndromic’ immunodeficiency may be underdiagnosed. To unequivocally prove this, a large case–control study would be needed; this is not really feasible. To explore this further, we performed a retrospective, observational survey study.

## Methods

An email message with the proposal to participate in a survey study was sent out to all members of the European Society for Immunodeficiencies (ESID) to identify as many patients known to ESID members as possible with a chromosomal aberration in combination with one or more identified immunological abnormalities relating to PID. Exclusion criteria were trisomy 21 (Down syndrome) and 22q11 deletion (DiGeorge syndrome), because the immunological abnormalities in these syndromes have been described in detail before [6, 7]. Those ESID members who agreed to participate in the study were requested to complete an online questionnaire for each of their eligible patients (Additional file 1). The patients were identified by physician recall. The answers to the questionnaires were encrypted and saved on a protected server; these data did not contain any information that enabled identification of the identity of the patients. Clinical characteristics and identified immunological abnormalities were reported. Age-matched reference values were used for interpretation of immunoglobulin levels and lymphocyte subpopulation counts; values below the age-matched reference values were scored as ‘low’ [29–31]. For the interpretation of the vaccine responses (i.e. before and after diagnostic vaccination with Tetanus and PneumoVax® or Pneumo(vax)23®) reference values from the laboratory performing the tests were used. For responses to Pneumovax® or Pneumo(vax)23® measured by serotype, a titer ≥1 IU/ml per serotype was considered to be a sufficient response. If only total IgG for S. pneumoniae was tested, a >4 fold increase of titer was considered as a positive response. Additional immunological tests were performed judged necessary by the treating physician and are therefore only available for some patients. Lymphocyte function tests included in vitro T lymphocyte proliferation tests (to Concanavalin A (ConA), phytohaemagglutinin (PHA), pokeweed mitogen (PWD) and Staphylococcus aureus enterotoxin A (SAE)), natural killer (NK) cell and cytotoxic T cell toxicity (in vitro stimulated CD107a degranulation). Granulocyte function tests included oxidative burst, the quantitative nitroblue tetrazolium dye reduction (NBT) test and phagocytosis test (cells Escherichia coli opsonised). For these additional immunological test (e.g. lymphocyte and granulocyte function tests), the laboratory-specific reference values were used. Furthermore, we asked all the participating centers to provide us with the number of patients with chromosomal aberrations who had undergone an immunological evaluation but were subsequently found *not* to have an immunological abnormality. This was also based on physician recall. Descriptive statistics were performed. The International System for Human Cytogenetic Nomenclature 2013 (ISCN) was used for cytogenetic nomenclature [32]; an overview is given as a group, and in relation to the specific chromosomal aberrations concerned. The Medical Ethical Committee Brabant approved of the study procedures.

## Results

Fifty-two patients from 16 different centers distributed globally were reported. Six patients had to be excluded because they did not meet the inclusion criteria: 3 patients did not have a confirmed chromosomal aberration, 1 patient with Rubinstein-Taybi Syndrome (no chromosomal aberration, only single gene mutation), 1 patient with suspected Kabuki Syndrome (no genetic diagnosis) and 1 patient with Rothmund-Thomson Syndrome (no chromosomal aberration, only single gene mutation). Three other patients did not have an immunodeficiency, these were 3 related patients with familial t (12;14). An overview of the excluded patients is presented in Additional file 2.

The 46 included patients consisted of 24 males and 22 females with a median age of 10.4 years at the moment of reporting (range 1.0–69.2 years; 36 pediatric and 10 adult patients). Two families were reported: patients 17 and 20 are related, as well as patients 18 and 19 (they are also related to the excluded patients 2, 3 and 4, see Additional file 2). Fifteen of these 46 included patients have been published before and publication of two patients is currently in press (for details see Table 1).

**Table 1:** Clinical characteristics of the included patients

Nr	Sex	Age (yrs)^1^	Genetics	Immunological presentation^2^	Other clinical presentations^3^	Other symptoms
1	M	15.2	46, XY, dup(6) (p12.2p21.31)	Airways	Developmental delay Dysmorphic features Microcephaly	Prematurity 36 weeks Tracheostomy Feeding difficulties Infantile pyloric stenosis Pulmonary congestion Intractable diarrhoea
2^(a)^	M	3.4	46, XY.ish der(16)t(16;19) (p13.3;p13.3) arr[hg19] 16p13.3(106 271–1 024 153)x1, 19p13.3 (327 273–6 887 622)x3	Failure to thrive	Developmental delay Ataxia, paresis or other motor disability Dysmorphic features Microcephaly Growth retardation	Bilateral inguinal hernia Horse shoe kidney Hypospadia, hydrocele Maldescensus testis
3^(a)^	M	9.4	46, XY.ish der(14)t(14;19) (p11.2;p13.2) de novo; arr[hg19] 19p13.3p13.2(90 897–7 300 043)x3	Unusual infections	Developmental delay Ataxia, paresis or other motor disability Dysmorphic features Microcephaly Growth retardation	Bilateral incarcerated inguinal hernia Congenital hip dysplasia Perineal hypospadia/penoscrotal fistula Severe osteopenia Sensorineurinal hearing loss Epilepsy
4	M	5.6	46, XY, del(18) (p11.2)	Airways	Ataxia, paresis or other motor disability Growth retardation	na
5	M	12.0	47, XY, +13	Airways	Developmental delay Growth retardation	Sepsis Seizures Gastroesophageal reflux disease Loss of vision
6	F	6.1	46, XX, del(16) (p11.2)	Airways	Developmental delay Dysmorphic features	Obesity Autism BCGosis
7	M	5.1	46, XY, del(2) (q33.2)	AI disease	Developmental delay Dysmorphic features	Cleft palate PDA Splenomegaly Auto-immune hemolytic anemia
8	M	1.0	No full karyotype available Array CGH : gain of 144kB in 9p24.3 and loss of 15MB in 10q26.11.q26.3	Unusual infections	Developmental delay Dysmorphic features Microcephaly Growth retardation	Duodenal atresia PDA Micropenis, gonadal agenesia
9	F	5.2	46, XX, del(18) (q22)	AI disease	Developmental delay Dysmorphic features	Auto-immune polyendocrine syndrome type II with: Thyroiditis Vitiligo Pernicious anemia Type 1 diabetes mellitus
10	F	1.4	46, XX, arr[hg19] 16p11.2 (29, 567, 295-30, 177, 916)x1 dn	Failure to thrive	Developmental delay Growth retardation	Recurrent fever
11	F	69.2	45, X	Airways	Dysmorphic features Growth retardation	Schwannoma Hearing loss
12	F	6.5	45, X[42]/47, XXX [8]	Airways	Developmental delay Growth retardation	Currarino syndrome
13	F	41.6	45, X	Unusual infections	Growth retardation	na
14	M	20.1	46, XY, der(X)t(X;18) (q28;q23) (MECP2 duplication)	Airways	Developmental delay Ataxia, paresis or other motor disability Dysmorphic features	Vitiligo Bronchiectasis Small intestinal villous atrophy
15^(b)^	M	7.5	46, XY, r(18)(p11.2q23) [97]/45, XY, -18 [3]	Airways	Developmental delay Ataxia, paresis or other motor disability Dysmorphic features Microcephaly Growth retardation	ASD II, VSD Micropenis
16^(c)^	F	47.7	arr[hg19] 11q24.2q25 (126, 074, 297-134, 927, 114)x1	Airways	Developmental delay Dysmorphic features Atopic eczema	VSD Infertility HPV associated giant condylomata Hypothyroid Idiopathic angio-edema Severe asthma Hypersplenism Obesity, type II diabetes Bronchiectasis
17^(d)^	M	22.3	46, XY, der(18)t(10p;18q) with 18q22.3–q23 deletion and partial trisomy of 10pter	Airways	Developmental delay Ataxia, paresis or other motor disability Dysmorphic features Growth retardation	Hypothyroid (subclinical) Pulmonary valve stenosis
18^(e)^	F	29.3	46, XX, t(12;14) (p11.2;q13)	AI disease	Atopic eczema	Samter's triad* ALL Migraine Recurrent herpes labialis HPV associated condylomata Multiple allergies
19^(e)^	F	4.9	46, XX, t(12;14) (p11.2;q13)	Airways	None	na
20^(d)^	F	28.2	46, XX, der(18)t(10p;18q) with 18q22.3–q23 deletion and partial trisomy of 10pter	Airways	Ataxia, paresis or other motor disability Dysmorphic features Atopic eczema	Thymus hyperplasia Atopy Polyarticular JIA
21	F	31.3	46, XX, arr[hg19] 15q25.2 (83, 214, 012-84, 776, 990)x1	Airways	None	Allergy Epilepsy Asthma Cholesteatoma Recurrent monoarthritis
22	M	34.3	46, XY, inv(10)(q21q23)	AI disease	None	Asymptomatic
23^(f)^	F	6.9	46, XX, del(19)(p13.13)	Airways	Developmental delay Ataxia, paresis or other motor disability Dysmorphic features Microcephaly Growth retardation	IUGR Epilepsy
24^(f)^	M	9.6	46, XY, r(18)	AI disease	Developmental delay Dysmorphic features Growth retardation Hypopigmentation	Panniculitis with lipodystrophy Auto-immune hypothyroidism Vitiligo Chronic urticaria Subaortic stenosis
25^(g)^	M	16.8	46, XY, der(11)dup(11) (q22q23)del(q24.3)	Airways	Developmental delay Ataxia, paresis or other motor disability Dysmorphic features Atopic eczema Hair and/or nail abnormalities	na
26	M	3.7	No full karyotype available arr[hg19]11p12-p11.12 (38.090.281-49.257.082)x1	Airways	Developmental delay Ataxia, paresis or other motor disability Dysmorphic features	Defective absorption folinic acid
27	F	10.3	46, XX, del(11)(q11)	Failure to thrive	Developmental delay Dysmorphic features Growth retardation Atopic eczema	na
28	F	5.9	49, XXXXX	Pyogenic infections	Developmental delay Ataxia, paresis or other motor disability Dysmorphic features	PS and ASD Hypermobility Radio-ulnar synosthosis
29	M	15.1	46, XY.ish del(X) (p11.3p11.3) (RP4-628F15+, RP11-245M24 dim, RP6-99M1-, RP4-689N3-, RP11-1409+)mat	Failure to thrive	Developmental delay Microcephaly Growth retardation	Visual impairment Retinitis pigmentosa
30	M	9.4	46, XY, r(6)	Airways	Developmental delay Dysmorphic features Microcephaly Growth retardation	Gastro-oesofageal reflux Dilated cardiomyopathy and small VSD
31^(h)^	F	12.8	46, XX, del(18)(p11.1)	Airways	Developmental delay Ataxia, paresis or other motor disability Dysmorphic features Growth retardation	Type I diabetes mellitus Growth hormone deficiency Autoimmune thyroiditis Pectus excavatum Retrognathia with absent maxillary chondyles
32	M	6.8	46, XY, del(7)(q22.3 q31.3)	Airways	Developmental delay Dysmorphic features	na
33	F	14.7	47, XX, +der(22)t(11;22) (q23;q11) mat (partial trisomy 11q)	Airways	Developmental delay Ataxia, paresis or other motor disability	Palatoschizis, preauricular tags Anus atresia Urolithiasis
34	F	7.9	46, XX.arr snp 2p23.1 (SNP_A-2078092->SNP_A-2248377)x1 mat	Same pathogen	Developmental delay Ataxia, paresis or other motor disability Dysmorphic features	Mitochondrial dysfunction Acracyanosis Bronchiectasis Hyposplenia
35	M	11.3	46, XY.arr[hg19] 3p14.3 (57, 994, 310-58, 071, 249)x1 pat	Airways	Developmental delay Dysmorphic features	Submucosal palatal schisis Transient neonatal macroglossi Hepatosplenomegaly
36	F	6.8	45, X	Airways	None	na
37	F	20.2	46, XX, der(2)t(2;10)(q37.3;q26.3)mat.arr snp 2q37.2q37.3(SNP_A-1957498->SNP_A-2027809)x1,10q26.3 (SNP_A-2264115->SNP_A-1934598)x3	Airways	Developmental delay Ataxia, paresis or other motor disability	Autistiform developmental delay Splenomegaly Cytopenias Granulomata Gastroparesis Obesitas
38^(i)^	M	6.5	49, XXXXY	Airways	Developmental delay Dysmorphic features	na
39^(i)^	M	10.6	49, XXXXY	Airways	Developmental delay Dysmorphic features	na
40^(i)^	M	14.6	49, XXXXY	Airways	None	na
41^(i)^	M	13.3	49, XXXXY	Airways	Developmental delay Dysmorphic features	na
42^(i)^	M	11.7	49, XXXXY	Airways	Developmental delay Dysmorphic features Atopic eczema	na
43	M	12.2	47, XYY, dup(22) (q11.21)	Pyogenic infections	Developmental delay Ataxia, paresis or other motor disability Dysmorphic features Microcephaly	Asthma
44	F	3.8	46, XX.arr snp 1q44 (SNP_A-2136114->SNP_A-4223408)x1 dn, 11p11.2p11.12 (SNP_A-1817808->SNP_A-4198132)x3 pat	Airways	Developmental delay Dysmorphic features Microcephaly Growth retardation Atopic eczema	Epilepsy Rocker bottom foot Cow’s milk allergy Feeding difficulties
45	F	6.7	46, X, idic(X) (p11.21).arr snp 22q11.21 (SNP_A-2108791->SNP_A-2160861)x3 mat, Xp22.33p11.21(SNP_A-4207883->2247707)x1 dn, Xp11.21q28(SNP_A-4201150->SNP_A-2267820)x3 dn	Airways	Developmental delay Growth retardation	Prematurity; gestational age 30 weeks Bone anchored hearing aid Periorbital hemangioma
46	F	12.9	46, XX, arr cgh 16p13.11p13.12 (14, 687, 636-16, 452, 200) x3.	Airways	Developmental delay Ataxia, paresis or other motor disability Dysmorphic features Microcephaly Growth retardation Atopic eczema	Severe scoliosis Seizures Myopathy of unknown etiology Chronic progressive external ophthalmoplegia Contractures; wheelchair bound

Headings: Nr = patient number; ^1^at the time of reporting; ^2^most prominent clinical immunological presentation; ^3^other clinical presentations as requested in the survey (Additional file 1)

Patients: (a) previously published in Seidel MG, Duerr C, Woutsas S, et al. J Med Genet 2014;51:254–263, (b) previously published in Celmeli F, et al. J Investig Allergol Clin Immunol. 2014;24(6):442–4, (c) previously published in Seppänen et al. J Clin Immunol 2014;34:114–118., (d) family members and previously published in Dostal et al. International Journal of Immu-genetics 2007;34: 143–147 : patient 17 as IV:4 and patient 20 as IV, (e) family members, together with excluded patient 2, 3 and 4, (f) publication in press, Calvo Campoverde K, et al. Allergologia et Immunopathologia 2016, (g) previously published in Fernandez-San Jose C, J Paediatr Child Health 2011;47(7):485–6, (h) previously published in Browning MJ, J Investig Allergol Clin Immu-l 2010;20(3):263–266, (i) previously published in Keller MD, et al. Am J Med Genet C Semin Med Genet. 2013;163C(1):50–4

Clinical presentations: Airways = Recurrent ENT and airway infections; FTT = failure to thrive from early infancy; unusual infections = unusual infections or unusually severe course of infections; AI disease = autoimmune or chronic inflammatory disease, lymphoproliferation; pyogenic infections = recurrent pyogenic infections; same pathogen = recurrent infections with the same type of pathogen (de Vries E. Clin Exp Immunol 2012;167(1):108–19.)

Other abbrevations: *ALL* acute lymphatic leukemia, *ASD* atrial septum defect, *BCG* Bacillus Calmette-Guérin, *F* female, *HPV* human papilloma virus, *IUGR* intra uterine growth retardation, *JIA* juvenile idiopathic arthritis, *M* male, *na* not available, *PDA* patent ductus arteriosus, *PEG* percutaneous endoscopic gastrostomy, *PS* pulmonary stenosis, *VSD* ventricular septum defect, yrs: years

* Samter’s triad: asthma, aspirin and NSAID sensitivity, and nasal/ethmoidal polyposis

Seven out of the total 16 centers provided the number of patients with chromosomal aberrations who had undergone an immunological evaluation but were subsequently found *not* to have an immunological abnormality. Together, they reported 27 patients with immunological abnormalities in this survey; they also reported 63 patients with chromosomal aberrations in whom immunological assessment revealed no abnormality. Thus, of these centers 30 % of the patients with chromosomal aberrations who underwent immunological evaluation were diagnosed with some form of primary immunodeficiency.

Symptoms indicative of PID can be divided into eight different clinical presentations [29]; ‘recurrent ear-nose-throat (ENT) and airway infections’ were most commonly reported in this cohort (in 43/46 patients). In 31/46 patients, ‘recurrent ENT and airway infections’ was reported as the clinically most important presentation. Other PID-related manifestations reported as the most important clinical presentation include ‘auto-immune or chronic inflammatory disease; lymphoproliferation’(*n* = 5); ‘failure to thrive from early infancy’ (*n* = 4); ‘unusual infections or unusually severe course of infections’(*n* = 3); ‘recurrent pyogenic infections’ (*n* = 2) and ‘recurrent infections with the same type of pathogen’(*n* = 1). The most common syndromic-related manifestations were: developmental delay (*n* = 37), ataxia, paresis or other motor disability (*n* = 16), dysmorphic features (*n* = 31), microcephaly (*n* = 11), growth retardation (*n* = 19), atopic eczema (*n* = 8), hair and/or nail abnormalities (*n* = 1) and hypopigmentation (*n* = 1). A detailed overview of the clinical findings is shown in Table 1.

**Fig. 1:**
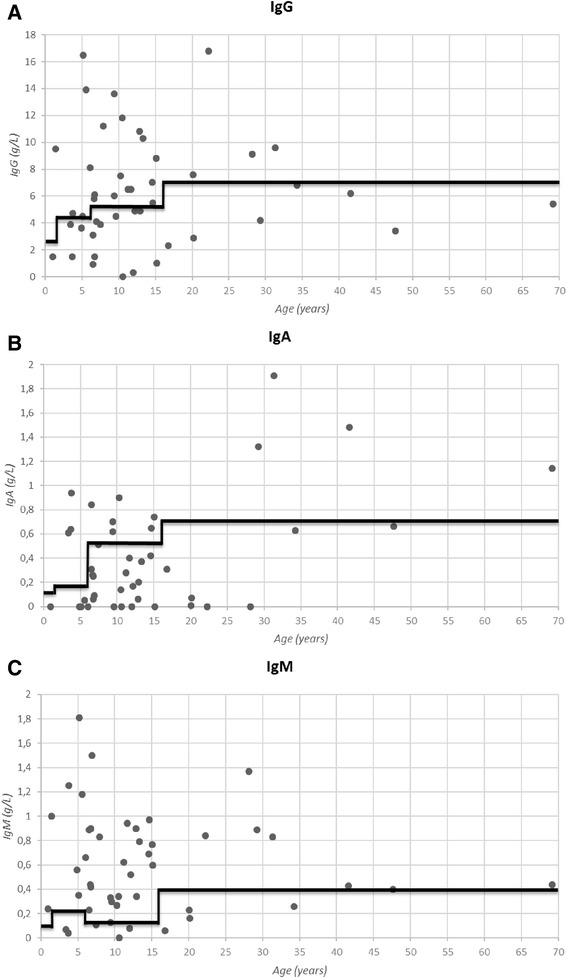
Levels of immunoglobulin isotypes. Every dot represents a patient. The bold black line is the lower limit of normal according to age-matched reference values (ref de Vries E. Clin Exp Immunol 2012;167(1):108–19.). **a**: IgG in g/L. **b**: IgA in g/L; two values > 2.0 g/l are not displayed in the graph. **c**: IgM in g/L

Antibody deficiency was the most common immunological defect identified. Of 33 patients reported to have low immunoglobulin isotype(s), 20 had low IgG (Fig. 1). Nine patients had low IgG with completely absent IgA and 8 patients had low IgG in combination with low IgM. IgG subclass deficiency was identified in 18 patients, of which 15 had concomitant low total immunoglobulin isotype(s) and 3 did not. Vaccine responses were tested in 32/46 patients and were found insufficient in 18 patients: 16/18 were insufficient for pneumococcal polysaccharide vaccine. Four patients (no. 2, 22, 23 and 24) showed normal antibody production after diagnostic vaccination despite low serum immunoglobulins. For patients 23 and 24, however, the decreased response was based on total IgG for S. pneumoniae. Responses to live vaccines were not documented; no unfavorable outcomes of natural chickenpox infection were reported. 18/46 patients were treated with immunoglobulin replacement. The indication of immunoglobulin replacement therapy was based on clinical grounds, as judged by the treating physician. In 2 patients lymphopenia was reported; one of them was neutropenic as well. Lymphocyte subpopulations (CD3, CD4, CD8, CD19 and CD16/56) were determined in 36 patients; in 16 a decreased count of ≥1 (sub)populations was reported. In 11 patients more extensive B cell subpopulations were determined and in 7 patients extensive T cell subpopulations (protocols differed per patient). In 1 patient total absence of B lymphocytes was reported (patient number 5; trisomy 13). Lymphocyte and granulocyte function tests were performed in 11 and 11 patients, respectively; in 2 decreased lymphocyte as well as granulocyte function was reported (patients 2 and 3). A detailed overview of the immunological and other laboratory findings is presented in Table 2 and Additional file 3.

**Table 2:** Results of immunological and other diagnostic tests in the included patients. A: Immunological screening tests

Nr	NP	LP	↓G	↓A	↓M	↓IgG subclass		Lymphocyte subsets	Resp TV	Resp P	L function	G function
1	-	-	+	+^*^	-	na		↑ aCD3, ↑ aCD3CD4	↓	↓	na	na
2^(a)^	-	-	+	-	+	+	IgG1, IgG3	↓ aCD3, ↓ a smB	nl	nl	↓ NK toxicity	Oxidative burst borderline ↓
											In vitro lymphocyte proliferation: nl	
3^(a)^	-	-	-	-	-	-		Borderline ↓ a smB	nl	nl	↓ vitro lymphocyte proliferation decreased from 7 years on: SEA	Moderate ↓ oxidative burst
4	-	-	-	+	-	na		na	na	nl	na	na
5	-	-	+	+^*^	+	+	IgG1, IgG2, IgG3	Absence of CD19 cells	na	na	In vitro lymphocyte proliferation: nl	na
6	-	-	-	+^*^	-	-		↑ aCD3, ↑ aCD3CD4, ↑ aCD19	nl	nl	na	nl
7	-	-	-	+^*^	-	na		↓ aCD3, ↓ aCD3CD8 ↓ aCD19	↓	na	na	na
8	-	-	+	+^*^	-	na		nl	na	na	na	na
9	-	-	-	-	-	-		↑ aCD16/56	↓	na	nl	na
10	-	-	-	-	-	na		↑ aCD3, ↑ aCD19, ↑ aCD16/56	nl	na	Thymic function: nl	na
11	-	-	+	-	-	na		↓ aCD3CD8, ↓ aCD19, ↑ aCD16/56	na	nl	na	nl
12	-	-	+	+	-	na		↑ aCD3, ↑ aCD3CD4, ↑ aCD19	nl	na	na	na
13	+	+	+	-	-	na		↓ aCD3CD4, ↓ aCD19, ↓ aCD16/56, ↑ aCD3CD8	na	na	na	na
14	-	-	-	+	+	+	IgG2, IgG4	nl	na	↓^(j)^	na	na
15^(b)^	-	-	-	-	+	-		nl	na	na	nl	nl
16^(c)^	-	-	+	+	-	+	IgG1, IgG2, IgG4	↓ aCD3, ↓ aCD3CD4, ↓ aCD19, ↓ aCD16/56	na	na	na	na
17^(d)^	-	-	-	+^*^	-	+^**^	IgG2, IgG4	nl	na	na	na	na
18^(e)^	-	-	+	-	-	+	IgG1, IgG2, IgG4	↓ aCD19, ↓ aCD16/56	na	na	na	na
19^(e)^	-	-	+	+^*^	-	+	IgG1	na	na	na	na	na
20^(d)^	-	-	-	+^*^	-	+	IgG4	↓ aCD16/56	na	na	na	na
21	-	-	-	-	-	+	IgG3, IgG4	↓ aCD19 cells, slightly ↓ aMZ-like B, ↑ aCD3, ↑ aCD3CD8	nl	nl	nl	na
22	-	+	+	+	+	-		↓ aCD3, ↓ aCD3CD4, ↓ aCD3CD8, ↓aCD19 cells (BM)	nl	nl	na	na
23^(f)^	-	-	+	+	-	+	IgG1, IgG2, IgG4	↓ aCD3CD4, ↓ aCD16/56	nl	nl^(j)^	↓ In vitro lymphocyte proliferation: PHA = 85 %, PWD = 72 %, ConA = 39 %	na
24^(f)^	-	-	+	+^*^	-	+^***^	IgG2, IgG3, IgG4	↓ aCD3CD8	nl	nl^(j)^	↓ In vitro lymphocyte proliferation: PHA = 92 %, PWD = 87 %, ConA = 28 %	na
25^(g)^	-	-	+	+	+	na		na	nl	↓	↓ In vitro lymphocyte proliferation: PHA	na
26	-	-	+	-	+	+	IgG1, IgG3	na	na	↓	na	na
27	-	-	-	-	-	-		↓ aCD3, ↓ aCD3CD4, ↓ aCD3CD8, ↓ aCD19, ↓ aCD16/56	↓	↓	↓ In vitro lymphocyte proliferation: PHA	na
28	-	-	-	+	+	na		↑ aCD3, ↑ aCD3CD4, ↑ aCD3CD8, ↑ aCD19	nl	nl	na	nl
29	-	-	-	-	-	-		↑ aCD3CD8, ↑ aCD19	nl	↓	na	nl
30	-	-	-	-	+	na		↓ aCD3, ↓ aCD3CD8 cells, ↑ aCD19	nl	↓	na	nl
31^(h)^	-	-	-	+	-	+	IgG2	↑ aCD3, ↑ aCD3CD4, ↑ aCD3CD8, ↑ aCD19	nl	↓	na	nl
32	-	-	-	+	+	na		↑ aCD19	nl	nl	na	nl
33	-	-	-	-	-	+	IgG1	na	na	nl	na	na
34	-	-	-	-	-	-		nl	na	↓	na	nl
35	-	-	-	+	-	+	IgG2	nl	na	↓	na	na
36	-	-	+	+	-	na		na	na	na	na	na
37	-	-	+	+	+	+	IgG1, IgG2	↓ aCD3, ↓ aCD3CD8, ↓ aCD19, ↓ aCD16/56 cells, ↓ a memB	na	na	na	na
38^(i)^	-	-	+	-	-	na		na	na	↓	na	na
39^(i)^	-	-	na	na	na	na		↑ aCD3CD4	na	↓	na	na
40^(i)^	-	-	-	-	-	na		↑ aCD3, ↑ aCD3CD4	na	↓	na	na
41^(i)^	-	-	-	-	-	na		na	na	↓	na	na
42^(i)^	-	-	-	-	-	-		↑ aCD3, ↑ aCD3CD4, ↑ aCD19, ↑ aCD16/56	na	↓	na	na
43	-	-	+	+	-	+	IgG2	↑ aCD3, ↑ aCD3CD4, ↑ aCD3CD8, ↑ aCD 19	nl	↓	na	na
44	-	-	-	-	-	+	IgG1	na	na	na	na	na
45	-	-	-	+	-	-		na	na	na	na	na
46	-	-	+	+	+	na		nl	na	na	na	na

Headings: *Nr* patient number, *NP* neutropenia, *LP* lymphopenia,↓*G*: low IgG,↓A: low IgA,↓M: low IgM.↓IgG subclass: low IgG subclasses, *Resp TV* response tetanus vaccine, *Resp P* response PneumoVax® or Pneumo23®, L function: lymphocyte function tests, G function: granulocyte function tests

Patients: (a) previously published in Seidel MG, Duerr C, Woutsas S, et al. J Med Genet 2014;51:254–263, (b) previously published in Celmeli F, J Investig Allergol Clin Immunol. 2014;24(6):442–4, (c) previously published in Seppänen et al. J Clin Immunol 2014;34:114–118., (d) family members and previously published in Dostal et al. International Journal of Immu-genetics 2007;34: 143–147 : patient 17 as IV:4 and patient 20 as IV, (e) family members, together with excluded patient 2, 3 and 4, (f) publication in press, Calvo Campoverde K, et al. Allergologia et Immunopathologia 2016, (g) previously published in Fernandez-San Jose C, J Paediatr Child Health 2011;47(7):485–6. (h) previously published in Browning MJ, J Investig Allergol Clin Immu-l 2010;20(3):263–266, (i) previously published in Keller MD, et al. Am J Med Genet C Semin Med Genet. 2013;163C(1):50–4, (j) decreased response to Pneumovax ® or Pneumo23® based on total IgG for S. pneumoniae

^*^ IgA completely absent, ^**^ IgG2 completely absent, ^***^IgG3 completely absent

Other abbrevations: *a* absolute cell count, *BM* bone marrow, *CD* cluster of differentiation, *ConA* Concanavalin A, *memB* memory B cells, *MZ* marginal zone, *na* not available, *nl* normal, *PHA* phytohaemagglutinin, *PWD* pokeweed mitogen, *SEA* Staphylococcus aureus enterotoxin A, *smB* switched memory B cells

## Discussion

Our call identified 46 patients with chromosomal aberration associated with immunodeficiency, the largest cohort reported in the literature so far (42 isolated cases, and twice 2 patients from the same family). Based on data from 7/16 participating centers, up to one third of patients with chromosomal aberrations and recurrent infections may have some form of primary immunodeficiency. Because the patients in this study were identified by physician recall, reporter bias is possible. However, the relative number is much higher than the 6 % found in a cohort of 259 ‘normal’ children screened for immunological abnormalities because of recurrent infections by Brodszki et al. [33]. The most common clinical presentation in our cohort was *‘*recurrent ENT and airway infections*’*, which triggered their physician to perform immunological investigations. Not surprisingly, these were mostly ‘predominantly antibody deficiencies’ [34] ranging from IgG-subclass deficiency and/or polysaccharide antibody deficiency to severe hypogammaglobulinemia or even agammaglobulinemia in one patient. While this study may have limitations inherent to a retrospective, observational survey (e.g. recall bias; reporting bias; convenience sampling), these findings suggest that 'syndromic immunodeficiency’ may be under-diagnosed.

A previous study in patients with dysmorphic features found low CD19^+^ and CD16^+^ and/or CD56^+^ cells as the most frequent immunological abnormalities, followed by low immunoglobulins [28]. However, in contrast to our survey this study also included a lot of patients with Down syndrome (11/29 patients) who are known to have lower CD19^+^ and CD3^−^CD16^+^ and/or CD56^+^ cells [6], precluding an appreciation for the possibility of underlying immunodeficiency in patients with non-Down, chromosomal syndromes. Until now, no other cohorts of patients with different chromosomal aberrations associated with immunological abnormalities have been described. The chromosomal aberrations described in our study may provide insight regarding novel genes involved in the immune system, either located directly within or adjacent to the anomalous loci. Several of the cytogenetic abnormalities in our patients have been linked to immunodeficiency or -dysregulation in the literature before.

The largest family in our cohort consists of 5 affected patients with 46, XX, t (12;14) (p11.2;q13) (patients 18, 19 and excluded patients 2, 3 and 4). Only two had immunodeficiency (patients 18 and 19), both with low IgG-levels and one with additional IgA-deficiency and decreased numbers of CD19^+^ and CD3^−^CD16^+^and/orCD56^+^ cells. All patients in this family suffered from atopy, asthma and/or allergy (some with anaphylaxis); two developed acute lymphatic leukemia. A candidate gene located on chromosome 14q13 is *nuclear factor of kappa light chain gene enhancer in B cells inhibitor alpha (NFKBIA)* (OMIM 164008). NFKBIA inactivates NF-kappa-B by trapping it in the cytoplasm. Functional impairment of NFKBIA can result in increased activation of the NF-kappa-B pathway leading to immune dysregulation [35].

The other family in our cohort consists of two cousins with an unbalanced translocation t (18q;10p), namely t (18q-;10p+) (patients 17 and 20), effectively resulting in a 18q22.3–q23 deletion and a partial trisomy of 10pter. Both showed IgA-deficiency and IgG-subclass deficiency (both IgG4 and one also IgG2), and one showed decreased numbers of CD3^−^CD16^+^and/or CD56^+^ cells. One of the cousins showed diffuse thymic hyperplasia (patient 20) without evidence of developing thymoma. Although patients with complete 10p trisomy are not reported to have immunodeficiency [36, 37], patients with terminal deletions of 10p have been reported with IgA- and IgG-deficiency before [21, 38]. The 18q − syndrome is associated with IgA-deficiency and other autoimmune or immunodeficiency diseases, such as common variable immunodeficiency (CVID) [39], juvenile rheumatic arthritis [40], insulin-dependent diabetes mellitus [41], celiac disease [42] and thyroid hormone abnormalities [43]. This partly matches the clinical phenotypes of our related patients. The other patients in our cohort with chromosome 18q aberrations all but one also showed IgA-deficiency (patients 9, 14, and 20). The two cousins from our study are part of a Finnish family with t (18q;10p), which was published in 2007 [20]. All members of this family showed IgA-deficiency; IgG-subclasses were not tested in the other family members. The authors hypothesized that the observed IgA-deficiency may result from haplo-insufficiency of one or multiple genes located in the 18q22.3–q23 region in possible connection with a larger polygenic network.

Our cohort contains two patients with ring chromosome 18 (one mosaic (patient 15) and one with complete chromosome 18 deletion (patient 24)) and one patient with 18p deletion (patient 31). Deletions of chromosome 18p have also been associated with immune-related dysfunction like autoimmune thyroiditis, diabetes mellitus, IgA deficiency, atopic skin conditions, juvenile rheumatoid arthritis [12, 15, 22, 25], and in one patient with SLE [12]. This matches with our patients: two patients had an IgA-deficiency and the patient with 18p deletion had multiple endocrine dysfunctions. However, our patient with a mosaic form of ring chromosome 18 (46, XY, r(18) (p11.2q23) [97]/45, XY, -18 [3], patient 15) showed only low IgM with recurrent respiratory tract infections, as published before [27].

Four of our patients showed chromosome 11q deletions (patients 16, 25, 27 and 33); two of them were published before [14, 26]. Terminal deletion of chromosome 11 can cause Jacobsen syndrome [44] and has previously been associated with hypogammaglobulinemia, pancytopenia and low T-helper cell counts [45, 46]. Our patients with 11q deletion did not show neutropenia or lymphopenia, but three of them had both IgG- and IgA-deficiency. No low T-helper cell counts were reported.

Two centers reported a patient with deletion of chromosome 16p11.2 (patients 6 and 10). Deletions in this region of chromosome 16 are associated with intellectual disability, congenital anomalies, obesity, macrocephaly, and autism [47]. This matches the clinical picture of one of our patients. Both patients showed only minor immunological abnormalities: IgA deficiency and global lymphocytosis. Recently, single nucleotide polymorphisms at the fused-in-sarcoma (FUS)/integrin CD11b (ITGAM) locus at 16p11.2 were associated with CVID phenotypes [48]. In the literature, there is also a report of an autistic girl with a 16p11.2 deletion who also had severe combined immunodeficiency (SCID) caused by Coronin-1A deficiency (also located at 16p11.2) [49]. Coronin-1A is essential for development of a normal peripheral T cell compartment in mice as well as men [33, 50]. However, this girl had, in contrast to our patient, next to the 16p11.2 deletion, also a 2 bp deletion of the Coronin-1A gene on the other (paternal) allele.

Several patients with X-chromosome aberration were included. Turner syndrome (45, X) is known to be associated with immunodeficiency [5, 8], but with different clinical presentations. Our four Turner patients (patients 11, 13 and 36, and patient 12 with mosaicism Turner) also showed a variety of immunological abnormalities. The relationship, if any, between the immune defects in Turner syndrome and those in established X-linked PID remains unknown. Additionally, 5 boys with 49, XXXXY (patients 38, 39, 40, 41 and 42) and 1 girl with 49, XXXXX (patient 28) were reported. The 49, XXXXX girl presented with pyogenic infections and low IgG and IgM levels, but with normal granulocyte levels and function. The 49, XXXXY boys all presented with ‘recurrent ENT and airway infections’, and they all showed impaired antibody responses to pneumococcal polysaccharide antigens, as was published before [11].

## Conclusion

This retrospective survey demonstrates that patients with chromosomal aberrations and recurrent infections may harbor underlying primary immunodeficiencies. By specifically excluding the syndromic immunodeficiencies associated with Down and DiGeorge syndromes, we showed that a diverse spectrum of chromosomal aberrations can be associated with immunological abnormalities. In our cohort antibody deficiency was the most prevalent; this is important because infectious complications can be prevented with early interventions like antibiotic prophylaxis or immunoglobulin replacement treatment in these patients. To assess whether this association is a truly causal relation, a large case–control study would be needed; this is not really feasible. And of course, our survey results do not negate other contributing factors (e.g. aspiration; abnormal anatomy) in the development of recurrent ENT and airway infections in these patients. Nonetheless, our findings suggest it is important to consider immunological investigations in patients with chromosomal aberration and recurrent infections.

## Abbreviations

ConA, concanavalin A; CVID, common variable immunodeficiency; ENT, ear-nose-throat; ESID, European society for immunodeficiencies; FUS, fused-in-sarcoma; ISCN, International System for Human Cytogenetic Nomenclature; ITGAM, integrin CD11b; NBT, nitroblue tetrazolium dye reduction; NFKBIA, nuclear factor of kappa light chain gene enhancer in B cells inhibitor alpha; NK, natural killer cell; PHA, phytohaemagglutinin; PID, primary immunodeficiency; PWD, pokeweed mitogen; SAE, staphylococcus aureus enterotoxin A; SCID, severe combined immunodeficiency

## Additional files

Additional file 1: The Online Questionnaire. (DOCX 20 kb) Additional file 2: Clinical and immunological characteristics of the excluded patients. (DOCX 24 kb) Additional file 3: Additional test results of the included patients. (DOCX 24 kb)

## References

[1] Shima H, Kitagawa H, Wakisaka M, Furuta S, Hamano S, Aoba T (2010). The usefulness of laryngotracheal separation in the treatment of severe motor and intellectual disabilities. Pediatr Surg Int.

[2] Yong PL, Boyle J, Ballow M, Boyle M, Berger M, Bleesing J, Bonilla FA, Chinen J, Cunninghamm-Rundles C, Fuleihan R (2010). Use of intravenous immunoglobulin and adjunctive therapies in the treatment of primary immunodeficiencies: a working group report of and study by the primary immunodeficiency committee of the American academy of allergy asthma and immunology. Clin Immunol.

[3] Quinti I, Soresina A, Guerra A, Rondelli R, Spadaro G, Agostini C, Milito C, Trombetta AC, Visentini M, Martini H (2011). Effectiveness of immunoglobulin replacement therapy on clinical outcome in patients with primary antibody deficiencies: results from a multicenter prospective cohort study. J Clin Immunol.

[4] Bousfiha A, Jeddane L, Al-Herz W, Ailal F, Casanova JL, Chatila T, Conley ME, Cunningham-Rundles C, Etzioni A, Franco JL (2015). The 2015 IUIS phenotypic classification for primary immunodeficiencies. J Clin Immunol.

[5] Ming JE, Stiehm ER, Graham JM (2003). Syndromic immunodeficiencies: genetic syndromes associated with immune abnormalities. Crit Rev Clin Lab Sci.

[6] Kusters MA, Verstegen RH, Gemen EF, de Vries E (2009). Intrinsic defect of the immune system in children with down syndrome: a review. Clin Exp Immunol.

[7] Davies EG (2013). Immunodeficiency in DiGeorge syndrome and options for treating cases with complete athymia. Front Immunol.

[8] Lorini R, Ugazio AG, Cammareri V, Larizza D, Castellazzi AM, Brugo MA, Severi F (1983). Immunoglobulin levels, T-cell markers, mitogen responsiveness and thymic hormone activity in Turner’s syndrome. Thymus.

[9] Hanley-Lopez J, Estabrooks LL, Stiehm R (1998). Antibody deficiency in wolf-hirschhorn syndrome. J Pediatr.

[10] Bart IY, Weemaes CM, Schuitema-Dijkstra AR, Smeets D, de Vries E (2011). Immunodeficiency in a child with partial trisomy 6p. Acta Paediatr.

[11] Keller MD, Sadeghin T, Samango-Sprouse C, Orange JS (2013). Immunodeficiency in patients with 49, XXXXY chromosomal variation. Am J Med Genet C: Semin Med Genet.

[12] McGoey RR, Gedalia A, Marble M (2011). Monosomy 18p and immunologic dysfunction: review of the literature and a new case report with thyroiditis, IgA deficiency, and systemic lupus erythematosus. Clin Dysmorphol.

[13] Seidel MG, Duerr C, Woutsas S, Schwerin-Nagel A, Sadeghi K, Neesen J, Uhrig S, Santos-Valente E, Pickl WF, Schwinger W (2014). A novel immunodeficiency syndrome associated with partial trisomy 19p13. J Med Genet.

[14] Seppanen M, Koillinen H, Mustjoki S, Tomi M, Sullivan KE (2014). Terminal deletion of 11q with significant late-onset combined immune deficiency. J Clin Immunol.

[15] Recalcati MP, Valtorta E, Romitti L, Giardino D, Manfredini E, Vaccari R, Larizza L, Finelli P (2010). Characterisation of complex chromosome 18p rearrangements in two syndromic patients with immunological deficits. Eur J Med Genet.

[16] Batanian JR, Braddock SR, Christensen K, Knutsen AP (2013). Combined immunodeficiency in a 3-year-old boy with 16p11.2 and 20p12.2-11.2 chromosomal duplications. Am J Med Genet A.

[17] Browning MJ (2010). Specific polysaccharide antibody deficiency in chromosome 18p deletion syndrome and immunoglobulin A deficiency. J Investig Allergol Clin Immunol.

[18] Balikova I, Vermeesch JR, Fryns JP, Van Esch H (2009). Bronchiectasis and immune deficiency in an adult patient with deletion 2q37 due to an unbalanced translocation t(2;10). Eur J Med Genet.

[19] Artac H, Reisli I, Yildirim MS, Bagci G, Luleci G, Hosgor O, Karaaslan S (2009). Hypogammaglobulinemia and silver-russell phenotype associated with partial trisomy 7q and partial monosomy 21q. Am J Med Genet A.

[20] Dostal A, Linnankivi T, Somer M, Kahkonen M, Litzman J, Tienari P (2007). Mapping susceptibility gene locus for IgA deficiency at del(18)(q22.3-q23); report of familial cryptic chromosome t(18q; 10p) translocations. Int J Immunogenet.

[21] Cingoz S, Bisgaard AM, Bache I, Bryndorf T, Kirchoff M, Petersen W, Ropers HH, Maas N, Van Buggenhout G, Tommerup N (2006). 4q35 deletion and 10p15 duplication associated with immunodeficiency. Am J Med Genet A.

[22] Sripanidkulchai R, Suphakunpinyo C, Jetsrisuparb C, Luengwattanawanich S (2006). Thai girl with ring chromosome 18 (46XX, r18). J Med Assoc Thai.

[23] Broides A, Ault BH, Arthus MF, Bichet DG, Conley ME (2006). Severe combined immunodeficiency associated with nephrogenic diabetes insipidus and a deletion in the Xq28 region. Clin Immunol.

[24] Imai K, Shimadzu M, Kubota T, Morio T, Matsunaga T, Park YD, Yoshioka A, Nonoyama S (2006). Female hyper IgM syndrome type 1 with a chromosomal translocation disrupting CD40LG. Biochim Biophys Acta.

[25] Kellermayer R, Gyarmati J, Czako M, Teszas A, Masszi G, Ertl T, Kosztolanyi G (2005). Mos 46, XX, r(18).ish r(18)(18ptel-,18qtel-)/46, XX.ish del(18)(18ptel-): an example for successive ring chromosome formation. Am J Med Genet A.

[26] Fernandez-San Jose C, Martin-Nalda A, Vendrell Bayona T, Soler-Palacin P (2011). Hypogammaglobulinemia in a 12-year-old patient with jacobsen syndrome. J Paediatr Child Health.

[27] Celmeli F, Turkkahraman D, Cetin Z, Mihci E, Yegin O (2014). Selective IgM deficiency in a boy with ring chromosome 18. J Investig Allergol Clin Immunol.

[28] Mahmoud SA, Lowery-Nordberg M, Chen H, Thurmon T, Ursin S, Bahna SL (2005). Immune defects in subjects with dysmorphic disorders. Allergy Asthma Proc.

[29] de Vries E (2012). Patient-centred screening for primary immunodeficiency, a multi-stage diagnostic protocol designed for non-immunologists: 2011 update. Clin Exp Immunol.

[30] Schatorje EJ, Gemen EF, Driessen GJ, Leuvenink J, van Hout RW, van der Burg M, de Vries E (2011). Age-matched reference values for B-lymphocyte subpopulations and CVID classifications in children. Scand J Immunol.

[31] Schatorje EJ, Gemen EF, Driessen GJ, Leuvenink J, van Hout RW, de Vries E (2012). Pediatric reference values for the peripheral T-cell compartment. Scand J Immunol.

[32] Shaffer JM-J LG, Schmid M (2013). ISCN (2013): an international system for human cytogenetic nomenclature.

[33] Brodszki N, Jonsson G, Skattum L, Truedsson L (2014). Primary immunodeficiency in infection-prone children in southern Sweden: occurrence, clinical characteristics and immunological findings. BMC Immunol.

[34] Al-Herz W, Bousfiha A, Casanova JL, Chatila T, Conley ME, Cunningham-Rundles C, Etzioni A, Franco JL, Gaspar HB, Holland SM (2011). Primary immunodeficiency diseases: an update on the classification from the international union of immunological societies expert committee for primary immunodeficiency. Front Immunol.

[35] Auphan N, DiDonato JA, Rosette C, Helmberg A, Karin M (1995). Immunosuppression by glucocorticoids: inhibition of NF-kappa B activity through induction of I kappa B synthesis. Science.

[36] Berend SA, Shaffer LG, Bejjani BA (1999). Pure trisomy 10p involving an isochromosome 10p. Clin Genet.

[37] Lozic B, Culic V, Lasan R, Tomasovic M, Samija RK, Zemunik T (2012). Complete trisomy 10p resulting from an extra stable telocentric chromosome. Am J Med Genet A.

[38] Stone D, Ning Y, Guan XY, Kaiser-Kupfer M, Wynshaw-Boris A, Biesecker L (1996). Characterization of familial partial 10p trisomy by chromosomal microdissection, FISH, and microsatellite dosage analysis. Hum Genet.

[39] Slyper AH, Pietryga D (1997). Conversion of selective IgA deficiency to common variable immunodeficiency in an adolescent female with 18q deletion syndrome. Eur J Pediatr.

[40] Rosen P, Hopkin RJ, Glass DN, Graham TB (2004). Another patient with chromosome 18 deletion syndrome and juvenile rheumatoid arthritis. J Rheumatol.

[41] Gordon MF, Bressman S, Brin MF, de Leon D, Warburton D, Yeboa K, Fahn S (1995). Dystonia in a patient with deletion of 18q. Mov Disord.

[42] Lipschutz W, Cadranel S, Lipschutz B, Martin L, Clees N, Martin JJ, Wauters JG, Coucke P, Willems P (1999). 18q-syndrome with coeliac disease. Eur J Pediatr.

[43] Schaub RL, Hale DE, Rose SR, Leach RJ, Cody JD (2005). The spectrum of thyroid abnormalities in individuals with 18q deletions. J Clin Endocrinol Metab.

[44] Mattina T, Perrotta CS, Grossfeld P (2009). Jacobsen syndrome. Orphanet J Rare Dis.

[45] Penny LA, Dell’Aquila M, Jones MC, Bergoffen J, Cunniff C, Fryns JP, Grace E, Graham JM, Kousseff B, Mattina T (1995). Clinical and molecular characterization of patients with distal 11q deletions. Am J Hum Genet.

[46] von Bubnoff D, Kreiss-Nachtsheim M, Novak N, Engels E, Engels H, Behrend C, Propping P, de la Salle H, Bieber T (2004). Primary immunodeficiency in combination with transverse upper limb defect and anal atresia in a 34-year-old patient with jacobsen syndrome. Am J Med Genet A.

[47] Miller DT NR, Sobeih MM, et al.: 16p11.2 Microdeletion. In*.* Edited by Pagon RA AM, Ardinger HH, et al.: GeneReviews® [Internet]. Seattle (WA): University of Washington, Seattle; 1993–2015. Available from: http://www.ncbi.nlm.nih.gov/books/NBK11167/; 2009 Sep 22 [Updated 2011 Oct 27].

[48] Maggadottir SM, Li J, Glessner JT, Li YR, Wei Z, Chang X, Mentch FD, Thomas KA, Kim CE, Zhao Y (2015). Rare variants at 16p11.2 are associated with common variable immunodeficiency. J Allergy Clin Immunol.

[49] Shiow LR, Paris K, Akana MC, Cyster JG, Sorensen RU, Puck JM (2009). Severe combined immunodeficiency (SCID) and attention deficit hyperactivity disorder (ADHD) associated with a coronin-1A mutation and a chromosome 16p11.2 deletion. Clin Immunol.

[50] Foger N, Rangell L, Danilenko DM, Chan AC (2006). Requirement for coronin 1 in T lymphocyte trafficking and cellular homeostasis. Science.

