# Supplementary material for: Primary immunodeficiency associated with chromosomal aberration – an ESID survey
Source: Orphanet J Rare Dis. 2016 Aug 2;11:110. doi: 10.1186/s13023-016-0492-1 (PMC4971718; doi:10.1186/s13023-016-0492-1)
Supplement: Additional file 1: — The Online Questionnaire. (DOCX 20 kb) [file 13023_2016_492_MOESM1_ESM.docx]

**Additional file 1: the Online Questionnaire**

**Primary immunodeficiency associated with chromosomal aberration – an ESID Survey**

Ellen Schatorjé^1^, MD, Michiel van der Flier^2^, MD, PhD, Mikko Seppänen^3^, MD, PhD, Michael Browning^4^, FRCPath, Megan Morsheimer^5^, MD, MPH, Stefanie Henriet^2^, MD, PhD, João Farela Neves^6^, MD, Donald Cuong Vinh^7^, MD, PhD, Laia Alsina^8^, MD, PhD, Anete Grumach^9^, MD, PhD, Pere Soler-Palacin^10^, MD, PhD, Thomas Boyce^11^, MD, Fatih Celmeli^12^, MD, Ekaterini Goudouris^13^, MD, PhD, Grant Hayman^14^, PhD, Richard Herriot^15^, FRCP, Elisabeth Förster-Waldl^16^, MD, PhD, Markus Seidel^17^, MD, Annet Simons^18^, PhD, Esther de Vries^1,19^, MD, PhD.

**Affiliations:** ^1^Dept Pediatrics, Jeroen Bosch Hospital, 's-Hertogenbosch, the Netherlands, ^2^Dept of Pediatrics, Amalia Children's Hospital and Radboud Institute for Molecular Life Sciences, Radboudumc, Nijmegen, the Netherlands, ^3^Immunodeficiency Unit, Inflammation Center and Center for Rare Diseases, Children’s Hospital, Helsinki University and Helsinki University Hospital, Finland, ^4^University Hospitals of Leicester NHS Trust, United Kingdom, ^5^Children's Hospital of Philadelphia, United States, ^6^ Primary Immunodeficiencies unit Hospital Dona Estefania, Centro Hospitalar de Lisboa Central, Lisbon, Portugal, ^7^McGill University Health Centre, Montreal, Canada, ^8^Allergy and Clinical Immunology Department, Hospital Sant Joan de Deu, Barcelona, Spain, ^9^Faculty of Medicine ABC, São Paulo, Brazil, ^10^Pediatric Infectious Diseases and Immunodeficiencies Unit. Hospital Universitari Vall d'Hebron. Barcelona, Spain, ^11^Mayo Clinic, Rochester, Minnesota, United States, ^12^Antalya Education and Research Hospital Department of Pediatric Immunology and Allergy, Turkey, ^13^Universidade Federal do Rio de Janeiro, Brazil, ^14^Epsom & St Helier University Hospitals NHS Trust, United Kingdom, ^15^NHS Grampian, Scotland, ^16 .^ Dept. of Pediatrics and Adolescent Medicine, Center for Congenital Immunodeficiencies, Medical University Vienna, Austria, ^17^Pediatric Hematology-Oncology, Medical University Graz, Austria, ^18^Department of Human Genetics, Radboudumc, Nijmegen, The Netherlands, ^19^Dept Tranzo, Tilburg University, Tilburg, the Netherlands.

**Email addresses:**

Ellen Schatorjé: [e.schatorje@alumni.maastrichtuniversity.nl](mailto:e.schatorje@alumni.maastrichtuniversity.nl);

Michiel van der Flier: [Michiel.vanderFlier@radboudumc.nl](mailto:Michiel.vanderFlier@radboudumc.nl);

Mikko Seppänen: [Mikko.Seppanen@hus.fi](mailto:Mikko.Seppanen@hus.fi);

Michael Browning: [michael.browning@uhl-tr.nhs.uk](mailto:michael.browning@uhl-tr.nhs.uk);

Megan Morsheimer: [Megan.morsheimer@nemours.org](mailto:Megan.morsheimer@nemours.org);

Stefanie Henriet: [s.henriet@cukz.umcn.nl](mailto:s.henriet@cukz.umcn.nl);

João Farela Neves: [jpfn13@gmail.com](mailto:jpfn13@gmail.com);

Donald Cuong Vinh: [donald.vinh@mcgill.ca](mailto:donald.vinh@mcgill.ca);

Laia Alsina: [lalsina@hsjdbcn.org](mailto:lalsina@hsjdbcn.org);

Anete Grumach: [asgrumach@gmail.com](mailto:asgrumach@gmail.com);

Pere Soler-Palacin^:^ [psoler@vhebron.net](mailto:psoler@vhebron.net);

Thomas Boyce: [Boyce.Thomas@mayo.edu](mailto:Boyce.Thomas@mayo.edu);

Fatih Celmeli: [fcelmeli@hotmail.com](mailto:fcelmeli@hotmail.com);

Ekaterini Goudouris: [egoudouris@gmail.com](mailto:egoudouris@gmail.com);

Grant Hayman: [Grant.Hayman@esth.nhs.uk](mailto:Grant.Hayman@esth.nhs.uk);

Richard Herriot: [richard.herriot@nhs.net](mailto:richard.herriot@nhs.net);

Elisabeth Förster-Waldl: [elisabeth.foerster-waldl@meduniwien.ac.at](mailto:elisabeth.foerster-waldl@meduniwien.ac.at);

Markus Seidel: [markus.seidel@medunigraz.at](mailto:markus.seidel@medunigraz.at);

Annet Simons: [Annet.Simons@radboudumc.nl](mailto:Annet.Simons@radboudumc.nl);

Esther de Vries: [e.d.vries@jbz.nl](mailto:e.d.vries@jbz.nl);

**Address correspondence to:** Prof. dr. Esther de Vries, MD, PhD, Department of Pediatrics, Jeroen Bosch Hospital, P.O. Box 90153, 5200 ME ‘s-Hertogenbosch, [e.d.vries@jbz.nl](mailto:e.d.vries@jbz.nl); [e.devries@tilburguniversity.edu](mailto:e.devries@tilburguniversity.edu), phone +31-73-5532458/2966**,** fax +31-73-5532948.

*I.* ***Physician*** *submitting the survey (one form per submitted patient)*

NB the reporting physician, not the patient!

This information will be used as author information for the manuscript.

1. family name ...

2. initials ...

3. titles ...

4. affiliation ...

5. email address ...

*II.* ***Patient*** *with chromosomal aberration and associated immunological abnormalities being*

*reported*

1. male/female

2. date of birth

3. date of report (today)

4. chromosomal aberration present not being Down syndrome (trisomy 21) or DiGeorge

syndrome (22q11 deletion): yes/no

5. please enter as full a description of the chromosomal aberration as possible

(example: 46,XY,dup(6)(p12.2p21.31) or when SNP array was performed please state brand and type of chip

used and SNP positions: example: Affymetrix 250k SNP 46,XX,der(2)t(2;10)(q37.3;q26.3)mat.arr snp

2q37.2q37.3(SNP_A-1957498->SNP_A-2027809)x1,10q26.3(SNP_A-2264115->SNP_A-1934598)x3 ):

..................

*III. Clinical characteristics of the reported* ***patient***

1. Which clinical presentations apply to the patient? (multiple answers possible)

a. Recurrent ENT and airway infections

b. Failure to thrive from early infancy

c. Recurrent pyogenic infections

d. Unusual infections or unusually severe course of infections

e. Recurrent infections with the same type of pathogen

f. Autoimmune or chronic inflammatory disease; lymphoproliferation

2. What is the **clinically most important** clinical presentation of the patient? (single answer):

a. Recurrent ENT and airway infections

b. Failure to thrive from early infancy

c. Recurrent pyogenic infections

d. Unusual infections or unusually severe course of infections

e. Recurrent infections with the same type of pathogen

f. Autoimmune or chronic inflammatory disease; lymphoproliferation

3. Does the patient suffer from (multiple answers possible):

a. developmental delay

b. ataxia, paresis or other motor disability

c. dysmorphic features

d. microcephaly

e. growth retardation

f. atopic eczema

g. hair and/or nail abnormalities

h. hypopigmentation

4. Any other relevant clinical information, specification of the above: ....

*IV. Immunological characteristics of the reported* ***patient***

1. Was a leukocyte differential performed (absolute numbers)? yes/no

2. if yes: granulocytes (10e9/l)

3. if yes: lymphocytes (10e9/l)

4. Were immunoglobulins (IgG, IgA, IgM) determined in serum? yes/no

5. if yes: level of IgG (g/l)

6. if yes: level of IgA (g/l)

7. if yes: level of IgM (g/l)

8. Were IgG-subclasses determined in serum? yes/no

9. if yes: level of IgG1 (g/l)

10. if yes: level of IgG2 (g/l)

11. if yes: level of IgG3 (g/l)

12. if yes: level of IgG4 (g/l)

13. Were lymphocyte subpopulations determined?

no / yes (percentage only) / yes (absolute number) (single answer)

14. if yes (%): CD3+ T-lymphocytes

15. if yes (%): CD3+CD4+ helper-T-lymphocytes

16. if yes (%): CD3+CD8+ cytotoxic T-lymphocytes

17. if yes (%): CD19+ or CD20+ B-lymphocytes

18. if yes (%): CD3- CD16 and/or CD56+ NK-cells

19. if yes (10e9/l): CD3+ T-lymphocytes

20. if yes (10e9/l): CD3+CD4+ helper T-lymphocytes

21. if yes (10e9/l): CD3+CD8+ cytotoxic T-lymphocytes

22. if yes (10e9/l): CD19+ or CD20+ B-lymphocytes

23. if yes (10e9/l): CD3- CD16 and/or CD56+ NK-cells

24. Were any other lymphocyte subpopulations determined? yes/no

if yes: please email or fax an anonymized copy of the results

25. Were vaccine response(s) determined? no / yes (tetanus) / yes (PneumoVax®,

Pneumo23®) / yes (other) (multiple answer)

if yes: please email or fax an anonymized copy of the results

26. Were any tests of granulocyte function performed? yes/no

if yes: please email or fax an anonymized copy of the results

28. Were any tests of lymphocyte function performed? yes/no

if yes: please email or fax an anonymized copy of the results

Thank you for returning this online survey!
