# Supplementary material for: Primary immunodeficiency associated with chromosomal aberration – an ESID survey
Source: Orphanet J Rare Dis. 2016 Aug 2;11:110. doi: 10.1186/s13023-016-0492-1 (PMC4971718; doi:10.1186/s13023-016-0492-1)
Supplement: Additional file 2: — Clinical and immunological characteristics of the excluded patients. (DOCX 24 kb) [file 13023_2016_492_MOESM2_ESM.docx]

**Additional File 2: Clinical and immunological characteristics of the excluded patients.**

**Primary immunodeficiency associated with chromosomal aberration – an ESID Survey**

Ellen Schatorjé^1^, MD, Michiel van der Flier^2^, MD, PhD, Mikko Seppänen^3^, MD, PhD, Michael Browning^4^, FRCPath, Megan Morsheimer^5^, MD, MPH, Stefanie Henriet^2^, MD, PhD, João Farela Neves^6^, MD, Donald Cuong Vinh^7^, MD, PhD, Laia Alsina^8^, MD, PhD, Anete Grumach^9^, MD, PhD, Pere Soler-Palacin^10^, MD, PhD, Thomas Boyce^11^, MD, Fatih Celmeli^12^, MD, Ekaterini Goudouris^13^, MD, PhD, Grant Hayman^14^, PhD, Richard Herriot^15^, FRCP, Elisabeth Förster-Waldl^16^, MD, PhD, Markus Seidel^17^, MD, Annet Simons^18^, PhD, Esther de Vries^1,19^, MD, PhD.

**Affiliations:** ^1^Dept Pediatrics, Jeroen Bosch Hospital, 's-Hertogenbosch, the Netherlands, ^2^Dept of Pediatrics, Amalia Children's Hospital and Radboud Institute for Molecular Life Sciences, Radboudumc, Nijmegen, the Netherlands, ^3^Immunodeficiency Unit, Inflammation Center and Center for Rare Diseases, Children’s Hospital, Helsinki University and Helsinki University Hospital, Finland, ^4^University Hospitals of Leicester NHS Trust, United Kingdom, ^5^Children's Hospital of Philadelphia, United States, ^6^ Primary Immunodeficiencies unit Hospital Dona Estefania, Centro Hospitalar de Lisboa Central, Lisbon, Portugal, ^7^McGill University Health Centre, Montreal, Canada, ^8^Allergy and Clinical Immunology Department, Hospital Sant Joan de Deu, Barcelona, Spain, ^9^Faculty of Medicine ABC, São Paulo, Brazil, ^10^Pediatric Infectious Diseases and Immunodeficiencies Unit. Hospital Universitari Vall d'Hebron. Barcelona, Spain, ^11^Mayo Clinic, Rochester, Minnesota, United States, ^12^Antalya Education and Research Hospital Department of Pediatric Immunology and Allergy, Turkey, ^13^Universidade Federal do Rio de Janeiro, Brazil, ^14^Epsom & St Helier University Hospitals NHS Trust, United Kingdom, ^15^NHS Grampian, Scotland, ^16 .^ Dept. of Pediatrics and Adolescent Medicine, Center for Congenital Immunodeficiencies, Medical University Vienna, Austria, ^17^Pediatric Hematology-Oncology, Medical University Graz, Austria, ^18^Department of Human Genetics, Radboudumc, Nijmegen, The Netherlands, ^19^Dept Tranzo, Tilburg University, Tilburg, the Netherlands.

**Email addresses:**

Ellen Schatorjé: [e.schatorje@alumni.maastrichtuniversity.nl](mailto:e.schatorje@alumni.maastrichtuniversity.nl);

Michiel van der Flier: [Michiel.vanderFlier@radboudumc.nl](mailto:Michiel.vanderFlier@radboudumc.nl);

Mikko Seppänen: [Mikko.Seppanen@hus.fi](mailto:Mikko.Seppanen@hus.fi);

Michael Browning: [michael.browning@uhl-tr.nhs.uk](mailto:michael.browning@uhl-tr.nhs.uk);

Megan Morsheimer: [Megan.morsheimer@nemours.org](mailto:Megan.morsheimer@nemours.org);

Stefanie Henriet: [s.henriet@cukz.umcn.nl](mailto:s.henriet@cukz.umcn.nl);

João Farela Neves: [jpfn13@gmail.com](mailto:jpfn13@gmail.com);

Donald Cuong Vinh: [donald.vinh@mcgill.ca](mailto:donald.vinh@mcgill.ca);

Laia Alsina: [lalsina@hsjdbcn.org](mailto:lalsina@hsjdbcn.org);

Anete Grumach: [asgrumach@gmail.com](mailto:asgrumach@gmail.com);

Pere Soler-Palacin^:^ [psoler@vhebron.net](mailto:psoler@vhebron.net);

Thomas Boyce: [Boyce.Thomas@mayo.edu](mailto:Boyce.Thomas@mayo.edu);

Fatih Celmeli: [fcelmeli@hotmail.com](mailto:fcelmeli@hotmail.com);

Ekaterini Goudouris: [egoudouris@gmail.com](mailto:egoudouris@gmail.com);

Grant Hayman: [Grant.Hayman@esth.nhs.uk](mailto:Grant.Hayman@esth.nhs.uk);

Richard Herriot: [richard.herriot@nhs.net](mailto:richard.herriot@nhs.net);

Elisabeth Förster-Waldl: [elisabeth.foerster-waldl@meduniwien.ac.at](mailto:elisabeth.foerster-waldl@meduniwien.ac.at);

Markus Seidel: [markus.seidel@medunigraz.at](mailto:markus.seidel@medunigraz.at);

Annet Simons: [Annet.Simons@radboudumc.nl](mailto:Annet.Simons@radboudumc.nl);

Esther de Vries: [e.d.vries@jbz.nl](mailto:e.d.vries@jbz.nl);

**Address correspondence to:** Prof. dr. Esther de Vries, MD, PhD, Department of Pediatrics, Jeroen Bosch Hospital, P.O. Box 90153, 5200 ME ‘s-Hertogenbosch, [e.d.vries@jbz.nl](mailto:e.d.vries@jbz.nl); [e.devries@tilburguniversity.edu](mailto:e.devries@tilburguniversity.edu), phone +31-73-5532458/2966**,** fax +31-73-5532948.

**Part A: Clinical characteristics of the excluded patients.**

| ***Nr*** | ***Sex*** | ***Age (yrs)^1^*** | ***Genetics*** | ***Immunological presentation^2^*** | ***Other clinical presentations^3^*** | ***Other symptoms*** |
| --- | --- | --- | --- | --- | --- | --- |
| Excl 1 | M | 39.4 | Rubinstein Taybi Syndrome  (mutation in CREBBP gene) | Airways | Developmental delay  Dysmorphic features  Microcephaly  Growth retardation |  |
| Excl 2* | F | 41.7 | 46,XX,t(12;14)(p11.2;q13) | Airways | Atopic eczema | Atopy, eczema, asthma, allergy and angioedema;  no immunodeficiency |
| Excl 3* | F | 16.8 | 46,XX,t(12;14)(p11.2;q13) | Airways | Atopic eczema | Atopy, asthma, allergy and anaphylaxis;  no immunodeficiency |
| Excl 4* | F | 13.7 | 46,XX,t(12;14)(p11.2;q13) | AI disease | Atopic eczema | ALL, atopy, asthma and allergy;  no immunodeficiency |
| Excl 5 | F | 14.8 | Kabuki syndrome  (no genetic diagnosis) | Airways | Developmental delay  Dysmorphic features  Growth retardation |  |
| Excl 6 | M | 4.6 | Rothmund Thomson Syndrome  (mutation in RECQL4 gene) | Unusual infections | Dysmorphic features  Growth retardation  Atopic eczema |  |

***Headings:***

*Nr= patient number;* *^1^at the time of reporting; ^2^most prominent immunological clinical presentation; ^3^ other clinical presentations as requested in the survey (Additional File 1).*

***Clinical presentations:***

*Airways = Recurrent ENT and airway infections; unusual infections = unusual infections or unusually severe course of infections; AI disease = autoimmune or chronic inflammatory disease; lymphoproliferation (de Vries 2012).*

***Patients:***

** family members together with included patients 18 and 19*

***Other abbreviations:***

*ALL: acute lymphatic leukemia, Excl: excluded patient,F: female, M: male, yrs: years.*

**Part B: Immunological characteristics of the excluded patients.**

| ***Nr*** | ***NP*** | ***LP*** | ***↓G*** | ***↓A*** | ***↓M*** | ***↓IgG subclass*** | | ***Lymphocyte subsets*** | ***Resp TV*** | ***Resp P*** | ***L function*** | ***G function*** |
| --- | --- | --- | --- | --- | --- | --- | --- | --- | --- | --- | --- | --- |
| Excl 1 | - | - | + | + | + | na |  | ↓ aCD16/56 | na | na | na | na |
| Excl 2* | - | - | - | - | - | - |  | na | na | na | na | na |
| Excl 3* | - | - | - | - | - | - |  | na | na | na | na | na |
| Excl 4* | - | - | - | - | - | - |  | na | na | na | na | na |
| Excl 5 | - | - | + | + | - | + | IgG1, IgG2, IgG4 | ↑ aCD3 cells, ↓ a sm and MZ B, ↑ CD21low B | nl | nl** | na | na |
| Excl 6 | - | - | + | + | - | na |  | ↓ aCD3, ↓aCD3CD4, ↓ aCD3CD8, ↓ aCD19, ↓ aCD16/56 cells | na | na | In vitro lymphocyte proliferation decreased: PHA, PWM, OKT3 | na |

***Headings:***

*Nr: patient number, NP: neutropenia, LP: lymphopenia,↓G: low IgG,↓A: low IgA,↓M: low IgM.↓IgG subclass: low IgG subclasses, Resp TV: response tetanus vaccine, Resp P: response PneumoVax® or Pneumo23®, L function: lymphocyte function tests, G function: granulocyte function tests.*

***Patients:***

** family members together with included patients 18 and 19, ** decreased response to Pneumovax ® or Pneumo23® based on total IgG for S. pneumoniae..*

***Other abbreviations:***

*a: absolute count, CD: cluster of differentiation, MZ: marginal zone, na: not available, nl: normal, PHA: phytohaemagglutinin, PWM: pokeweed mitogen, OKT3: a monoclonal IgG2 antibody which binds the ε component of the CD3 signal-transduction complex.*
