# Supplementary material for: Primary immunodeficiency associated with chromosomal aberration – an ESID survey
Source: Orphanet J Rare Dis. 2016 Aug 2;11:110. doi: 10.1186/s13023-016-0492-1 (PMC4971718; doi:10.1186/s13023-016-0492-1)
Supplement: Additional file 3: — Additional test results of the included patients. (DOCX 24 kb) [file 13023_2016_492_MOESM3_ESM.docx]

**Additional File 3: Additional test results of the included patients.**

**Primary immunodeficiency associated with chromosomal aberration – an ESID Survey**

Ellen Schatorjé^1^, MD, Michiel van der Flier^2^, MD, PhD, Mikko Seppänen^3^, MD, PhD, Michael Browning^4^, FRCPath, Megan Morsheimer^5^, MD, MPH, Stefanie Henriet^2^, MD, PhD, João Farela Neves^6^, MD, Donald Cuong Vinh^7^, MD, PhD, Laia Alsina^8^, MD, PhD, Anete Grumach^9^, MD, PhD, Pere Soler-Palacin^10^, MD, PhD, Thomas Boyce^11^, MD, Fatih Celmeli^12^, MD, Ekaterini Goudouris^13^, MD, PhD, Grant Hayman^14^, PhD, Richard Herriot^15^, FRCP, Elisabeth Förster-Waldl^16^, MD, PhD, Markus Seidel^17^, MD, Annet Simons^18^, PhD, Esther de Vries^1,19^, MD, PhD.

**Affiliations:** ^1^Dept Pediatrics, Jeroen Bosch Hospital, 's-Hertogenbosch, the Netherlands, ^2^Dept of Pediatrics, Amalia Children's Hospital and Radboud Institute for Molecular Life Sciences, Radboudumc, Nijmegen, the Netherlands, ^3^Immunodeficiency Unit, Inflammation Center and Center for Rare Diseases, Children’s Hospital, Helsinki University and Helsinki University Hospital, Finland, ^4^University Hospitals of Leicester NHS Trust, United Kingdom, ^5^Children's Hospital of Philadelphia, United States, ^6^ Primary Immunodeficiencies unit Hospital Dona Estefania, Centro Hospitalar de Lisboa Central, Lisbon, Portugal, ^7^McGill University Health Centre, Montreal, Canada, ^8^Allergy and Clinical Immunology Department, Hospital Sant Joan de Deu, Barcelona, Spain, ^9^Faculty of Medicine ABC, São Paulo, Brazil, ^10^Pediatric Infectious Diseases and Immunodeficiencies Unit. Hospital Universitari Vall d'Hebron. Barcelona, Spain, ^11^Mayo Clinic, Rochester, Minnesota, United States, ^12^Antalya Education and Research Hospital Department of Pediatric Immunology and Allergy, Turkey, ^13^Universidade Federal do Rio de Janeiro, Brazil, ^14^Epsom & St Helier University Hospitals NHS Trust, United Kingdom, ^15^NHS Grampian, Scotland, ^16 .^ Dept. of Pediatrics and Adolescent Medicine, Center for Congenital Immunodeficiencies, Medical University Vienna, Austria, ^17^Pediatric Hematology-Oncology, Medical University Graz, Austria, ^18^Department of Human Genetics, Radboudumc, Nijmegen, The Netherlands, ^19^Dept Tranzo, Tilburg University, Tilburg, the Netherlands.

**Email addresses:**

Ellen Schatorjé: [e.schatorje@alumni.maastrichtuniversity.nl](mailto:e.schatorje@alumni.maastrichtuniversity.nl);

Michiel van der Flier: [Michiel.vanderFlier@radboudumc.nl](mailto:Michiel.vanderFlier@radboudumc.nl);

Mikko Seppänen: [Mikko.Seppanen@hus.fi](mailto:Mikko.Seppanen@hus.fi);

Michael Browning: [michael.browning@uhl-tr.nhs.uk](mailto:michael.browning@uhl-tr.nhs.uk);

Megan Morsheimer: [Megan.morsheimer@nemours.org](mailto:Megan.morsheimer@nemours.org);

Stefanie Henriet: [s.henriet@cukz.umcn.nl](mailto:s.henriet@cukz.umcn.nl);

João Farela Neves: [jpfn13@gmail.com](mailto:jpfn13@gmail.com);

Donald Cuong Vinh: [donald.vinh@mcgill.ca](mailto:donald.vinh@mcgill.ca);

Laia Alsina: [lalsina@hsjdbcn.org](mailto:lalsina@hsjdbcn.org);

Anete Grumach: [asgrumach@gmail.com](mailto:asgrumach@gmail.com);

Pere Soler-Palacin^:^ [psoler@vhebron.net](mailto:psoler@vhebron.net);

Thomas Boyce: [Boyce.Thomas@mayo.edu](mailto:Boyce.Thomas@mayo.edu);

Fatih Celmeli: [fcelmeli@hotmail.com](mailto:fcelmeli@hotmail.com);

Ekaterini Goudouris: [egoudouris@gmail.com](mailto:egoudouris@gmail.com);

Grant Hayman: [Grant.Hayman@esth.nhs.uk](mailto:Grant.Hayman@esth.nhs.uk);

Richard Herriot: [richard.herriot@nhs.net](mailto:richard.herriot@nhs.net);

Elisabeth Förster-Waldl: [elisabeth.foerster-waldl@meduniwien.ac.at](mailto:elisabeth.foerster-waldl@meduniwien.ac.at);

Markus Seidel: [markus.seidel@medunigraz.at](mailto:markus.seidel@medunigraz.at);

Annet Simons: [Annet.Simons@radboudumc.nl](mailto:Annet.Simons@radboudumc.nl);

Esther de Vries: [e.d.vries@jbz.nl](mailto:e.d.vries@jbz.nl);

**Address correspondence to:** Prof. dr. Esther de Vries, MD, PhD, Department of Pediatrics, Jeroen Bosch Hospital, P.O. Box 90153, 5200 ME ‘s-Hertogenbosch, [e.d.vries@jbz.nl](mailto:e.d.vries@jbz.nl); [e.devries@tilburguniversity.edu](mailto:e.devries@tilburguniversity.edu), phone +31-73-5532458/2966**,** fax +31-73-5532948.

| ***Nr*** | ***Lymphocyte subpopulations (x10^e^9/l)*** | ***Additional immunological/hematological findings*** | | ***Other laboratory*** | ***MRI*** |
| --- | --- | --- | --- | --- | --- |
| 1 | CD3 2.19, CD3 1.70, CD8 0.52, CD19 0.50, CD16/56 0.15 | na | | na | na |
| 2^(a)^ | CD3 1.21, CD4 1.70, CD8 0.20, CD19 0.77, CD16/56 0.16 | NK degranulation:↓ | MBL ↓; CH50/AH50 nl | Alpha-Thalassemia Trait | Multiple diffuse hyperintense white matter lesions, pronounced atrophy, hydrocephalus, microcysts basal ganglia |
| 3^(a)^ | CD3 3.78, CD4 1.85, CD8 1.43, CD19 2.64, CD16/56 0.29 | NK degranulation: ↓ | MBL ↓; CH50/AH50 nl | na | Hypoplastic inferior vermis, sligthly extended temporal lobe |
| 4 |  | na | | na | na |
| 5 |  | na | | na | na |
| 6 | CD3 3.05, CD4 1.91, CD8 0.94, CD19 1.97, CD16/56 0.40 | na | | na | na |
| 7 | CD3 1.03, CD4 0.72, CD8 0.32, CD19 0.29, CD16/56 0.10 | Auto-immune anemia | | na | na |
| 8 | CD3 4.09, CD4 2.76, CD8 1.26, CD19 0.58, CD16/56 0.46 | na | | na | na |
| 9 | CD3 1.70, CD4 0.93, CD8 0.54, CD19 0.46, CD16/56 0.74 | Hypergammapathy, pernicious anemia | | na | na |
| 10 | CD3 5.60, CD4 3.20, CD8 2.00, CD19 3.40, CD16/56 0.63 | Lymphocytosis | | na | na |
| 11 | CD3 0.74, CD4 0.56, CD8 0.16, CD19 0.10, CD16/56 0.44 | na | | na | na |
| 12 | CD3 3.69, CD4 2.78, CD8 0.91, CD19 1.48, CD16/56 0.34 | na | | na | na |
| 13 | CD3 2.26, CD4 0.43, CD8 1.80, CD19 0.05, CD16/56 0.02 | Monocytopenia | | na | na |
| 14 | CD3 0.99, CD4 0.46, CD8 0.41, CD19 0.24, CD16/56 0.11 | na | | na | na |
| 15^(b)^ |  | Isohemagglutinin titers 1:1 | | na | Corpus callosum hypoplasia, delayed myelinisation |
| 16^(c)^ | CD3 0.37, CD4 0.09, CD8 0.27, CD19 0.02, CD16/56 0.05 | Chronic Paris-Trousseau type thrombocytopenia | | na | na |
| 17^(d)^ | CD3 0.80, CD4 0.51, CD8 0.02, CD19 0.29, CD16/56 0.16 | na | | na | na |
| 18^(e)^ | CD3 1.41, CD4 0.89, CD8 0.42, CD19 0.14, CD16/56 0.09 | na | | na | Occult subarachnoidal cyst left frontal lobe |
| 19^(e)^ |  | na | | na | na |
| 20^(d)^ | CD3 0.96, CD4 0.67, CD8 0.28, CD19 0.24, CD16/56 0.07 | na | | na | Demyelinating white matter lesions |
| 21 | CD3 1.94, CD4 1.10, CD8 0.97, CD19 0.15, CD16/56 0.28 | na | | Hypergonadotropic hypogonadism  Primary hypophosphatasia |  |
| 22 | CD3 0.60, CD4 0.33, CD8 0.16, CD19 0.07, CD16/56 0.12 | Thrombocytopenia | | na | na |
| 23^(f)^ | CD3 2.16, CD4 1.20, CD8 0.80, CD19 0.53, CD16/56 0.12 | na | | na | Hypoplasia corpus callosum, ventriculomegaly |
| 24^(f)^ | CD3 1.16, CD4 0.80, CD8 0.26, CD19 0.26, CD16/56 0.26 | na | | na | na |
| 25^(g)^ | CD3 0.72, CD4 0.46, CD8 0.21, CD19 0.06, CD 16/56 0.07 | Thrombocytopenia | | na | na |
| 26 |  | na | | na | na |
| 27 | CD3 0.53, CD4 0.25, CD8 0.27, CD19 0.11, CD16/56 0.09 | na | | na | na |
| 28 | CD3 3.08, CD4 1.98, CD8 1.15, CD19 0.91, CD16/56 0.31 | na | | na | na |
| 29 | CD3 1.96, CD4 1.08, CD8 0.82, CD19 0.59, CD16/56 0.32 | na | | na | na |
| 30 | CD3 0.86, CD4 0.61, CD8 0.22, CD19 0.84, CD16/56 0.24 | na | | na | na |
| 31^(h)^ | CD3 2.57, CD4 1.35, CD8 1.11, CD19 0.66, CD16/56 0.15 | na | | na | Low myelinisation |
| 32 | CD3 2.79, CD4 1.66, CD8 1.00, CD19 0.77, CD16/56 0.21 | na | | na | na |
| 33 |  | na | | na | na |
| 34 |  | na | | na | na |
| 35 |  | na | | na | na |
| 36 |  | na | | na | na |
| 37 |  | na | | na | na |
| 38^(i)^ |  | na | | na | na |
| 39^(i)^ | CD3 1.97, CD4 1.50, CD8 0.37, CD19 0.35, CD16/56 0.22 | na | | na | na |
| 40^(i)^ | CD 3 2.68, CD4 1.93, CD8 0.66, CD19 0.60, CD16/56 0.78 | na | | na | na |
| 41^(i)^ |  | na | | na | na |
| 42^(i)^ | CD3 22.70, CD4 15.60, CD8 0.65, CD19 3.97, CD16/56 1.70 | na | | na | na |
| 43 | CD3 2.61, CD4 1.57, CD8 0.87, CD19 1.26, CD 16/56 0.35 | na | | na | na |
| 44 |  | na | | na | na |
| 45 | CD3 1.70, CD4 0.80, CD8 0.80, CD19 0.20, CD16/56 0.55 | na | | na | na |
| 46 | CD3 1.35, CD4 0.81, CD8 0.54, CD19 0.45, CD16/56 0.18 | na | | na | na |

***Patients:***

*(a) previously published in Seidel MG, Duerr C, Woutsas S, et al. J Med Genet 2014;51:254-263, (b)* *previously published in Celmeli F, J Investig Allergol Clin Immunol. 2014;24(6):442-4, (c) previously publised in Seppänen et al. J Clin Immunol 2014;34:114–118., (d) family members and previously published in Dostal et al. International Journal of Immu-genetics 2007;34: 143–147 : patient 17 as IV:4 and patient 20 as IV, (e) family members, together with excluded patient 2, 3 and 4, (f) publication in press, Calvo Campoverde K, et al. Allergologia et Immunopathologia 2016, (g) previously published in Fernandez-San Jose C, J Paediatr Child Health 2011;47(7):485-6. (h) previously published in Browning MJ, J Investig Allergol Clin Immu-l 2010;20(3):263-266, (i) previously published in Keller MD, et al. Am J Med Genet C Semin Med Genet. 2013;163C(1):50-4.*

***Other abbrevations:***

*AH: alternative complement, CD: cluster of differentiation, CH: classical complement, MBL: mannose binding ligand, na: not available, nl: normal.*
